# Endodontic therapy and Medication-related osteonecrosis of the jaw onset: a scoping review and expert opinion-based qualitative meta-synthesis

**DOI:** 10.1186/s12903-025-06741-5

**Published:** 2025-08-23

**Authors:** R. Mauceri, M. Coppini, V. C. A. Caponio, F. Zamparini, C. Prati, G. Campisi

**Affiliations:** 1 Department of Precision Medicine in Medical, Surgical and Critical Care, University of Palermo, Palermo, Italy; 2 Department of Rehabilitation, fragility and continuity of care, Unit of Oral Medicine and Dentistry for fragile patients, University Hospital Palermo, Palermo, Italy; 3 Department of Biomedical and Dental Sciences and Morphofunctional Imaging, University of Messina, Messina, Italy; 4 Department of Clinical and Experimental Medicine, University of Foggia, Foggia, Italy; 5 Department of Biomedical and Neuromotor Sciences, Endodontic Clinical Section, School of Dentistry, University of Bologna, Bologna, Italy; 6 Department of Biomedicine, Neuroscience and Advanced Diagnostics (Bi.N.D), University of Palermo, Palermo, Italy

**Keywords:** Endodontic failure, Root canal therapy, Osteonecrosis of the jaw, Bisphosphonate-Associated osteonecrosis of the jaw, BRONJ, Medication-Related osteonecrosis of the jaw, MRONJ, Review

## Abstract

**Introduction:**

Medication-related osteonecrosis of the Jaw (MRONJ) is a serious side effect of bone-modifying agents (BMAs), including bisphosphonates, denosumab, and antiangiogenic drugs. Some dental procedures are recognized as local risk factors for MRONJ. Given the lack of specific guidelines for root canal therapy in patients treated with BMA, this scoping review aimed to investigate the risk of MRONJ associated with failed root canal therapy. Subsequently, a meta-synthesis of expert opinions was performed to identify existing recommendations and pitfalls of root canal therapy in patients at risk of MRONJ.

**Methods:**

A review search was conducted in PubMed, Scopus, and Web of Science to answer the PIO question: Is root canal therapy failure associated with an increased risk of developing MRONJ? Subsequently, a meta-synthesis was conducted to analyze guidelines, recommendations, and expert opinions regarding root canal therapy in BMA-treated patients.

**Results:**

Five studies were included. All studies were case reports and case series, except one case-control study. Twenty-three patients who developed MRONJ after root canal therapy failure were analyzed. Also, a case- control study including 65 patients receiving BMAs and 46 patients never treated with BMAs was evaluated. In the last one only 4.8% of patients under BMAs developed MRONJ. The most common BMA used was zoledronate. A total of 223 teeth underwent root canal therapy.

**Conclusions:**

This study highlights the potential risks of root canal therapy failure in BMA-treated patients, emphasizing the need to evaluate treatment appropriateness and risk-benefit ratio carefully.

## Introduction

Medication-Related Osteonecrosis of the Jaw (MRONJ) has been defined as an “adverse drug reaction described as the progressive destruction and death of bone that affects the mandible and maxilla of patients exposed to the treatment with medications known to increase the risk of the disease, in the absence of a previous radiation treatment” [1]. MRONJ is a potentially serious side effect associated with bone-modifying agents (BMAs), including bisphosphonates (BPs) and denosumab (DNB), and antiangiogenic drugs (AA), such as tyrosine kinase inhibitors [2].

In recent years, the categories of patients at risk of MRONJ have gradually changed due to the introduction of new drugs on the market and the approval of new indications for drugs already in use. MRONJ is usually reported in four groups of patients [3]:

- cancer patients with bone metastases (BM) or with multiple myeloma, usually receiving high doses of BMAs (HD-BMA);
- patients affected by Giant Cell Tumour of Bone, receiving a monthly injection of DNB (HD-BMAs);
- patients affected by breast cancer (BC) or prostate cancer (PC), without BM and under hormonal therapy, who receiving low doses of BMAs (LD-BMAs), at the same dosage of osteometabolic patients, to prevent Cancer Treatment-Induced Bone Loss (CTIBL) [2];
- osteometabolic patients under LD-BMAs.

MRONJ is a multifactorial disease for which etiology is not fully understood [1, 4]. Several risk factors have been reported associated with an increased risk of MRONJ, distinguishable as medication-related, systemic, and local risk factors [5]. The only type of risk factors that can be controlled and managed by oral care specialists are the local ones. The main local risk factors of MRONJ include dental, periodontal, and peri-implant infection, tooth extraction, dental implant, dentoalveolar surgery, ill-fitting dentures, and anatomical variations [1, 6–9].

In the past, tooth extractions were avoided as they were considered a major risk factor for MRONJ onset [7, 10]; for this reason, root canal therapy was considered a good option to postpone or eradicate the need for invasive dental procedures of tooth extraction [11–14].

By adopting this approach, the natural tooth is preserved, thereby avoiding surgery; however, it also maintains a potential source of infection, increasing the MRONJ risk [7, 15].

The novelty is that, in recent years, tooth extractions have been reported not to increase the risk of MRONJ onset in patients receiving BMAs if the procedures are performed safely [7, 8, 13]^15^– [17].

Moreover, some cases of MRONJ triggered by root canal therapy failure were reported [18–20].

Considering this change in protocols and perceived MRONJ risks, there are no specific indications regarding root canal therapy in patients undergoing BMA therapy.

Therefore, the present scoping review was performed to identify and analyze any specific guidelines for performing root canal therapy in patients undergoing BMA therapy and any related MRONJ risk; subsequently, a meta-synthesis based on expert opinions was also elaborated.

## Materials and methods

### Protocol

This scoping review was conducted based on the indications of the PRISMA protocol for scoping reviews [21, 22].

The study was developed on the PIO question:

P: Patients assuming low doses or high doses of BMAs.

I: Root canal therapy failure.

O: Occurrence of MRONJ.

The scoping review was based on the following research question: “Is root canal therapy failure associated with an increased risk of developing MRONJ?”

### Research strategy

Records were identified using different search engines (e.g., Medline/PubMed, Scopus and Web of Science) and by scanning reference lists of articles. For the search strategy, MeSH terms and free text words were combined through Boolean operators as follows: (“osteonecrosis of the jaw” OR “ONJ” OR “MRONJ” OR “Medication-related osteonecrosis of the jaw” OR “Bisphosphonate-Associated Osteonecrosis of the Jaw” OR “BRONJ”) AND (“endodontics” OR “Root Canal Therapy” OR “Dental Pulp Devitalization” OR “pulpitis” OR “pulpal disease” OR “apical infection” OR “periapical disease” OR “endodontic surgery” OR “apical surgery”). The research of the records and their selection was conducted by two authors (MC and RM), supported by a 3rd author (GC), with the role of resolving doubtful and conflicting situations. The research was completed in July 2024.

### Eligibility criteria

The inclusion criteria for the studies were as follows, such as human studies; published in English language, in which patients, administered of BMAs, underwent root canal therapy, and later occurrence of MRONJ development was recorded.

The exclusion criteria were as follows: studies that did not specify the type of BMA therapy (e.g., LD-BMAs vs. HD-BMAs) or the underlying diseases (e.g., cancer patients with BM or multiple myeloma vs. osteoporotic patients or cancer patients undergoing LD-BMAs for CTIBL), studies that included patients who underwent radiotherapy to the head and neck area, systematic reviews or meta-analysis, and studies published in a language other than English.

### Data extraction

Two independent reviewers (MC and RM) screened the retrieved studies assessed their eligibility based on the inclusion criteria and extracted data using a standardized data extraction template. The following data were screened:

- Name of the first author, year of publication, name of the journal, and study design.
- The total number of participants, age and sex.
- Primary disease, Type of BMAs, Duration of BMA therapy, Type of devitalized tooth, MRONJ onset, follow-up period.
- Instrumentation protocol, obturation protocol.
- Evaluated outcomes and measures employed.

In case of incomplete or missing information, the corresponding author of the papers was contacted for clarification. Any discrepancies were resolved through discussion or consultation with a third reviewer (GC).

### Meta-synthesis

A meta-synthesis was conducted to analyse the current recommendations, guidelines, and expert opinions regarding the management of MRONJ [23]. For the search strategy, MeSH terms and free text words were combined through Boolean operators as follows: (“Medication-Related Osteonecrosis of the Jaw” OR “MRONJ” OR “Bisphosphonate-Related Osteonecrosis of the Jaw” OR “osteonecrosis of the jaw”) AND (“guidelines” OR “recommendations” OR “expert opinion” OR “position paper” OR “consensus”). The research of the records and their selection was conducted by two authors (MC and RM), supported by a 3rd author (GC), with the role of resolving doubtful and conflicting situations. The research was completed in September 2024. Comprehensive search was performed in relevant databases to identify existing guidelines, expert consensus statements, and recommendations that addressed the topic of root canal treatment in patients receiving BMA therapy. These studies were screened for relevance, and only those directly addressing the clinical management of root canal therapy in this specific patient population were included in the meta-synthesis. The qualitative findings from each source were then summarized and compared, providing an overview of the recommended practices, risk assessments, and treatment protocols [24, 25]. The synthesis aimed to highlight commonalities and discrepancies between the different guidelines and expert opinions, with particular emphasis on the safety, efficacy, and best practices for root canal therapy in BMA-treated patients [26].

## Results

### Study selection and data collection process

The last search yielded 197 results (PubMed = 67, Scopus = 85, Web of Science = 45). These references were integrated into the EndNote reference software tool (Endnote X9.3.2, Clarivate Analytics).

**Fig. 1:**
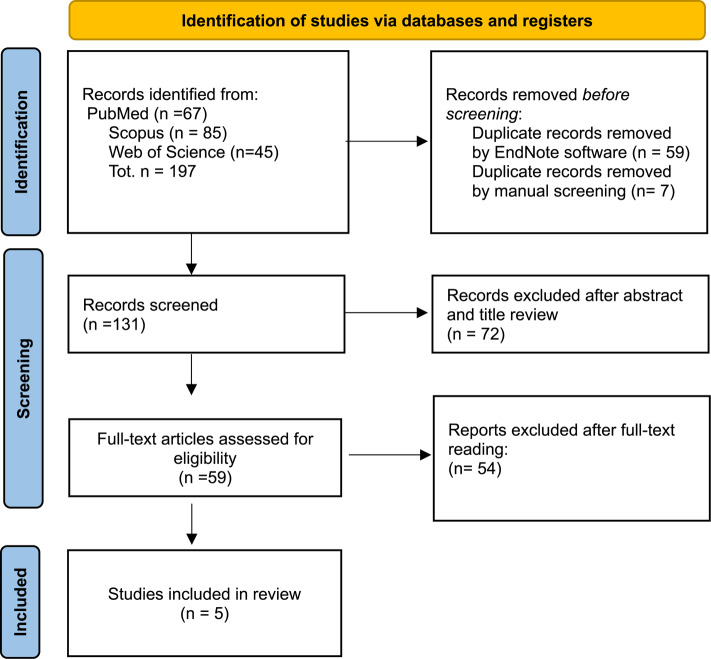
PRISMA 2020 flow chart

The initial search strategy identified 197 records, of which 66 were removed as they were duplicates. The screened process of 131 studies was performed based on the title and abstract, and 72 records were excluded. Subsequently, a full-text evaluation of 59 studies was carried out. Finally, based on the inclusion criteria 54 records were excluded, and 5 papers were included in the current review; a detailed flow chart of the selection process is provided in Fig. 1.

### Scoping review

Five studies were included in this review. The main characteristics of the selected studies are described in Table 1.

**Table 1:** Main characteristic of included studies

*N*	Author, Year	Country	*N*. of case	Age	Sex	Primary disease	Type of BMAs	Duration of BMA therapy	Type of devitalized tooth	MRONJ onset	Onset (time after root canal therapy)
1	Sarathy AP, 2005 [19]	USA	2	72 74	M	Prostate cancer with BM	#1: iv zoledronate #2: oral alendronate combined with iv pamidronate followed by iv zoledronate	#1: 15 months; #2: alendronate for 52 months, pamidronate for 14 months, and zoledronate for 27 months	#1: 3.7; #2: 1.5	Yes	n.d.
2	Gallego L, 2011 [27]	Spain	1	54	F	Multiple myeloma	zoledronate	19 months	3.7	Yes	5 months
3	Kaptan F, 2013 [18]	Turkey	2	62 64	F	Breast cancer with BM	#1: oral alendronate followed by iv zoledronate; #2: iv zoledronate	#1: oral alendronate for 48 months and iv zoledronate for 1 month; 2#: 48 months	#1: 3.5, 4.5, 47.; #2: 4.6	Yes	2 months
4	Zamparini F, 2021 [28]	Italy	Case – control Study (65 − 46)	BP group: 65.7 ± 8.6; ctr group: 60.3 ± 7.2	BP group: 52 F/5 M Ctr group: 21 F/25 M	29 cancer 36 osteometabolic disease	Alendronate: 21; clodronate: 8; risedronate: 1; zolendronate: 27; neridronate: 2; ibadronate: 6	22 to140 months	BP group: anterior: 24, premolarars: 31, molars: 41; control group: anterior: 20, premolars: 40, molars: 42	Yes 2 (4.8%)	60 months
5	Tempesta A, 2023 [20]	Italy	18	64.3 ± 3	12 F/6 M	Bladder cancer: 3; breast cancer: 7; prostate cancer: 4; multiple myeloma: 4	zoledronate: 10; clodronate: 3; denosumab: 4; bevacizumab: 1	23.27 months	n.d.	Yes	n.d.

*Acronyms: BP *bisphosphonates, *n.d.* not determined

All the included articles were observational studies published between 2005 and 2023. In detail, one case report [27], three case series [18–20] and one case-control study [28] were included. Of the five studies, two were from Italy [20, 28], one from the USA [19], one from Spain [27], and one from Turkey [18].

A total of 23 patients from the case reports and series developed MRONJ [18–20, 27]. A case-control study, performed by Zamparini et al., was included; it analyzed 65 patients assuming BMAs and 46 without ever taking BMAs, both underwent root canal therapy [28]. In this study, 8 patients of the BP group failed to comply with follow-up and were excluded [28].

The age of patients was not specified in all studies; based on the available data, in the study performed by Zamparini et al., the mean age was 65.7 ± 8.6 and 60.3 ± 7.2 years in the BP and control group, respectively [28], in the study performed by Tempesta et al. the mean age was 64.3 ± 3 [20] and the mean age of the rest of the patients included was 65.2 ± 7.2 years [18, 19, 27].

Regarding the primary disease, only one study included also patients affected by osteometabolic disease [28]. In detail, of the patients taking BMAs, 52 were cancer patients (52/88, 59.1%), and 36 were osteoporotic patients (36/88, 40.9%). Based on available data, among cancer patients, 9 were affected by breast cancer, 6 by prostate cancer, 5 by multiple myeloma, and 3 by bladder cancer. Unfortunately, in the study performed by Zamparini F et al., the type of cancer was not specified [28].

Regarding BMA therapy, the most common treatment was intravenous zoledronate (42/88, 47.7%), alone or in combination with oral alendronate, followed by alendronate (21/88, 23.9%), clodronate (11/88, 12.5%), ibandronate (6/88, 6.8%), neridronate (2/88, 2.3%), risedronate (1/88, 1.1%), denosumab (4/88, 4.5%), and bevacizumab (1/88, 1.1%).

Duration of BMA therapy was Heterogeneous. Zoledronate therapy was administered for a mean duration of 27.3 ± 14.7 months [18, 19, 27], one patient assumed zoledronate for 27 months after taking alendronate for 52 months and pamidronate for 14 months [19] and one patient assumed zoledronate for 1 month after taking alendronate for 48 months [18]. The mean duration of BMA therapy was not reported in two studies [20, 28]. Still, the overall duration of BMA therapy was reported, which in the study performed by Zamparini et al. ranged between 22 to140 months [28] and in the study performed by Tempesta et al., the mean duration of BMA and/or AA therapy was 23.27 months [20].

A total of 223 teeth underwent root canal therapy. Regarding the type of teeth that underwent root canal therapy, only 4 studies reported it. Three studies reported the specific teeth, which in two cases were 3.7 and the others 1.5, 3.5, 4.5, 4.7 and 4.6 respectively [18, 19, 27]. One study indicated that 44 of devitalized teeth were anterior teeth, 71 premolars, and 83 molars [28]. Unfortunately, in the study conducted by Tempesta et al., it was not specified the type of teeth that underwent root canal therapy [20]. Type of root canal treatment was specified in four out of five studies, namely 168 primary root canal treatments and 37 secondary root canal treatments [18, 19, 27, 28]. One study (18 teeth) did not report the type of treatment [20].

Regarding the endodontic procedure, in two studies the instrumentation technique was specified; in detail, in one study it was performed with rotary files and in one with manual k files [18, 28]. The same studies specified the filling material employed, which was gutta-percha (GP) with AH plus, Thermafil (TF), or GP with AH plus, respectively [18, 28, 29].

The follow-up period was respectively at least 7, 9, 12, and 60 months in included studies [18, 19, 27, 28]. In one study, the follow-up was not reported [20].

In the case-control study, only 4.8% of patients under BMAs developed MRONJ [28].

In the case report and series, all included patients developed MRONJ after root canal therapy failure. These patients were affected by cancer (highest MRONJ risk), and most were treated with zoledronate. Instead, in the case-control study performed by Zamparini F et al., in which only 4.8% of patients developed MRONJ, 55.4% of included patients were affected by osteometabolic disease (lower MRONJ risk). Moreover, in this study, teeth with insufficient structure for functional restoration were restored with gingival tissue-level restoration after root canal treatment to prevent occlusal stress and to permanently seal the root canals [28].

In no studies, other comorbidities different from those already mentioned (cancer vs. osteometabolic disease), nor local risk factors were reported.

Among the potential endodontic trigger factors of MRONJ, there were root canal overfilling, underfilling, root perforation, and root fracture caused by endodontic pin [20]. Only one study did not report a problem related to the failure of endodontic therapy, but rather to rubber dam clamp trauma [27].

The time between root canal therapy and MRONJ onset ranged from 2 to 60 months [18, 27, 28]. In two studies the MRONJ onset time was not reported [19, 20].

The most common clinical signs and symptoms of MRONJ reported in the studies were bone exposure, ulceration, tooth mobility, purulent drainage, pain, and extra-oral fistulas [18–20, 27].

Regarding reporting adverse drug reactions (ADRs), no study specified whether it was reported to regulatory authorities [30].

### Meta-synthesis

After a comprehensive search and screening to identify existing guidelines, expert consensus statements, and recommendations that addressed the topic of root canal treatment in patients receiving BMA therapy, 3 studies were included [1, 31, 32]. In all other studies, the risk associated with root canal therapy in patients undergoing BMA treatment is not mentioned, nor there were any indications for its implementation.

In detail, one study was the Italian Position Paper [1], one study was the American Association of Oral and Maxillofacial Surgeons’ Position Paper [31], and one was the Multinational Association of Supportive Care in Cancer/International Society of Oral Oncology (MASCC/ISOO) and ASCO Clinical Practice Guideline [32].

These studies address the management of endodontic treatment in patients exposed to BMAs, with some differences.

The American Association of Oral and Maxillofacial Surgeons acknowledges the unknown risk of MRONJ in patients who underwent root canal therapy, recommending conservative management with crown removal and retention of the root for non-restorable teeth [31]. The Multinational Association of Supportive Care in Cancer and the International Society of Oral Oncology Clinical Practice Guidelines advocate for either root canal therapy or tooth extraction for severely compromised teeth, without predilection [32]. In contrast, the Italian Position Paper emphasizes endodontic treatment specifically for teeth with good prognosis in patients on LD or HD- BMAs [1].

## Discussion

The present review, to the best of our knowledge, analyzed for the first time the existing literature data regarding root canal therapy failure in patients under BMA therapy.

To date, some studies have investigated the ideal dental management for patients at risk of MRONJ; however, there is limited evidence on the different approaches for treating odontogenic infections or on specific dental conservative procedures [33].

Endodontic treatment was previously considered the treatment of choice to control odontogenic infection and avoid tooth extraction in patients under BMA therapy [14, 33].

According to a study performed in patients not taking BMAs, nonsurgical endodontic treatment appears to be a relatively safe procedure for preserving natural dentition, with demonstrated high rates of survival and long-term success [34].

Nevertheless, in the last years, in patients assuming BMAs some cases of MRONJ after root canal therapy failure have been reported.

In our opinion, a thorough analysis of the criteria for endodontic therapy in patients at risk of MRONJ is essential since, under normal conditions (in the absence of BMA therapy), failure of root canal therapy may lead to tooth loss, but in patients taking BMAs it could also be a trigger for MRONJ.

In the last decade, it has been demonstrated that tooth extraction during BMA therapy does not increase the risk [7, 15]. According to a recent national cohort-based study, the risk of MRONJ increases significantly when tooth extraction is performed in patients diagnosed with periodontal disease, the latter representing a risk of MRONJ [8]. Also, Marques-Ferreira M. et al. reported that tooth extraction with a pre-existing periapical lesion increases the probability of MRONJ development [15, 17].

Therefore, it is essential to investigate and identify any precautions to be taken to perform “MRONJ-safe” root canal therapy in this category of patients and any indications and contraindications of such a procedure.

Regarding antibiotic prophylactic therapy, although there is no clear consensus on its use before nonsurgical root canal treatment in patients under bisphosphonate treatment, some authors recommend it in selected high-risk cases, including patients on intravenous BPs, those on long-term oral BPs (more than 3 years), or requiring multiple endodontic treatments [35].

Additionally, some precautions have been suggested to be taken during root canal therapy in patients under BMAs to reduce the risk of MRONJ, such as avoiding patency or instrumentation beyond the apex (27). However, adequate apical patency is essential for the effective elimination of microorganisms, as it provides access to the foraminal surface and periapical area and ensures the distribution of chemical solutions and intracanal drugs throughout the length of the root canal. Since endodontic and periapical lesions are often colonized by various microorganisms, incomplete permeabilization of the tooth apex may contribute to potential treatment failure [17].

Moreover, under normal conditions in the absence of BMA therapy, after root canal treatment, apical lesions usually heal through a process of bone remodeling, but in the case of bisphosphonate therapy, this process would be inhibited [35]. The lack of bone remodeling delays the healing process and, if associated with microleakage or incomplete sealing, could increase the risk of inflammation, thus also increasing the risk of MRONJ onset [36]. Furthermore, failure of endodontic treatment may cause periapical infection; in this condition, local inflammation, the bone resorption microenvironment, and the compromised blood flow in the periapical lesions may contribute to the onset of MRONJ in predisposed patients undergoing BMA therapies [17].

Moreover, Gallego et al. reported a case of MRONJ following trauma caused by a rubber dam clamp during endodontic treatment, suggesting that mucosal, periodontal, and alveolar bone trauma related to the clamp positioning during conservative procedures may represent a risk factor for MRONJ [27].

Another issue is the challenges in the diagnosis since, in some cases, the clinical and radiological features of root canal therapy failure and MRONJ can be similar (e.g., purulent drainage, periapical radiolucency, pain). This overlap in clinical and radiological signs may lead to misdiagnosis, as distinguishing between these conditions based on clinical and radiological findings alone can be challenging [16]. So, in the absence of specific signs and symptoms, often leading to clinical misdiagnoses, a detailed medical history and a meticulous clinical and radiological evaluation through second-line CT-based imaging (MDCT/CBCT) are mandatory in patients taking BMAs [37].

From our literature analysis, it emerges that root canal therapy is not free from failure, both in the long and short term.

Some studies, performed in patients without BMA therapy, have also examined possible prognostic factors of endodontic-periodontal lesions to evaluate the results of success, survival, and failure. The result was that bone loss in the apical third and probing depths of 5–7 mm and 8–10 mm were associated with a significantly lower probability of success and the absence of periodontal disease with a statistically significantly higher probability of success [38]. Another study evaluated potential predictors of early failure of endodontic treatment, including 175 patients undergoing endodontic treatment, and the result was that the presence of untreated additional canals was a predictor of endodontic failure within 5 years following initial root canal treatment [39].

Concerning BMA therapy and root canal therapy, based on the available data, the mean duration of zoledronate therapy of patients included in the present scoping review was 27.3 ± 14.7 months [18, 19, 27]. According to a study performed by Dereci Ö et al., which compared the clinical and radiographic success of non-surgical endodontic therapy in patients receiving intravenous zoledronate for less than 1 year and more than 1 year, a strong relationship was observed between the duration of bisphosphonate therapy and endodontic success [40].

Regarding potential confounding factors that can influence the MRONJ onset, in the included studies, no comorbidities (e.g., chronic kidney disease, diabetes mellitus, rheumatoid arthritis, etc.) or other local risk factors (e.g., periodontitis, ill-fitting dentures, etc.) were reported.

To the best of our knowledge, there are few prospective studies of patients on BMA therapy undergoing root canal therapy [28, 29]; therefore, it is not possible to assess a robust likelihood of MRONJ onset after failed root canal therapy. Furthermore, there are several cases reported in the literature that are cases of MRONJ following failed root canal therapies.

Although there are several cases reported in the literature are cases of MRONJ following failed root canal therapies, they are limited to case reports or case series; thus, due to the small sample size, the obtained results cannot be generalized, which limits the robustness of our conclusions. For this reason, and to address an important gap in the literature by exploring the potential risks of root canal therapy in patients at risk of MRONJ, we conducted this scoping review, supplemented by a meta-synthesis of expert opinions.

According to the American Association of Oral and Maxillofacial Surgeons, the risk of developing MRONJ among patients who have been exposed to BMAs for other dentoalveolar operations, including endodontic procedures, is unknown [31]. In their Position Paper, Ruggiero SL et al. suggested that non-restorable teeth can be treated by removal of the crowns and endodontic treatment of the remaining root [31].

According to the Multinational Association of Supportive Care in Cancer and the International Society of Oral Oncology Clinical Practice Guideline, teeth with severe caries, pulp involvement, or dental abscess, should undergo root canal therapy or tooth extraction, without discrimination regarding when to perform one therapy or the other [32].

Based on the Italian position paper on MRONJ, endodontic treatment is indicated in patients assuming LD or HD-BMAs to treat emerging dental diseases in teeth with good prognosis [1].

Recently, a review of endodontic treatment criteria was published by Ferrari et al. [33]. They have analyzed treatment criteria in patients at risk of MRONJ. According to them, in cases of odontogenic infections, the risks and benefits of various treatment approaches, (conservative or invasive) must be carefully evaluated and shared with the patient at MRONJ risk. In their manuscript, they recommended clinical protocols for the management of dental infections in patients at risk of developing MRONJ [33]. As reported by Ferrari et al., in the case of patients with good oral hygiene and small and localized periapical lesions (< 0.5 cm), a conservative approach is advisable. On the contrary, there are few contraindications for endodontic treatment, including the presence of periapical lesions > 0.5 cm or that require apicoectomy, acute dental abscess, and significant systemic comorbidities such as uncontrolled diabetes. In these cases, tooth extraction can definitively remove the focus of infections [33].

Our paper has some limitations derived from the heterogeneity of the few studies currently in the literature, mostly case series, in which some data are missing (e.g., specific indications for performing endodontic therapy or the results of blood tests). The limited number of articles available could be explained by two variables: the absence of an established association between endodontic failure and MRONJ onset and the lack of awareness of clinical and radiological findings of MRONJ; this condition also emerges from the meta-synthesis. Moreover, since most of the included studies were case reports or case series describing MRONJ following failed root canal therapy, this may introduce a selection bias, as only one case-control study included patients who underwent root canal treatment without developing MRONJ. For this reason, a scoping review was conducted instead of a systematic review to map the available evidence and highlight existing gaps in the literature. An expert opinion-based qualitative meta-synthesis was performed to address these limitations and provide greater scientific robustness to our study.

Finally, in the authors’ opinion, cases of MRONJ defined by some authors as “spontaneous” since reported in the absence of such risk factors, could be attributable to the failure of root canal therapy; this would lead to underestimating the failure of endodontic therapy as a risk factor for MRONJ [32, 41].

Based on the literature analysis, there is no consensus on the criteria for MRONJ-safe endodontic treatment in patients treated with BMAs. In this regard, future studies are needed to provide step-by-step protocols for root canal therapy management in high-risk patients from diagnosis to treatment and follow-up.

Most of the included studies agree that the prognosis of the tooth determines the most appropriate treatment.

In the case of endodontic diseases, in patients at risk of MRONJ, when a conservative approach is not possible and a good prognosis of the tooth is not guaranteed, the extraction of the affected tooth is the most recommended procedure to reduce the risk of MRONJ [5, 15].

## Conclusion

MRONJ is a potentially serious adverse drug reaction that can significantly impact patients’ quality of life if not promptly diagnosed and managed. This study represents the first attempt to investigate the data in the literature regarding the failure of root canal therapy and MRONJ development. Since root canal therapy does not appear to be a risk-free therapy for MRONJ, before performing root canal therapy in patients taking BMAs, its appropriateness must be meticulously evaluated, always evaluating the risk-benefit ratio.

## Data Availability

Not applicable.
